# Evaluation of a brief intervention within a stepped care whole of service model for personality disorder

**DOI:** 10.1186/s12888-019-2308-z

**Published:** 2019-11-06

**Authors:** Elizabeth Huxley, Kate L. Lewis, Adam D. Coates, Wayne M. Borg, Caitlin E. Miller, Michelle L. Townsend, Brin F. S. Grenyer

**Affiliations:** 1 School of Psychology and Illawarra Health and Medical Research Institute, University of Wollongong, Wollongong, NSW 2522 Australia; 2 South East Sydney Local Health District, Sydney, Australia

**Keywords:** Personality disorder, Borderline personality disorder, Model of care, Stepped care, Brief intervention, Crisis intervention, Suicide prevention

## Abstract

**Background:**

Although there is growing evidence that stepped models of care are useful for providing appropriate, person centered care, there are very few studies applied to personality disorders. A brief, four session, psychological treatment intervention for personality disorder within a whole of service stepped care model was evaluated. The intervention stepped between acute emergency crisis mental health services and longer-term outpatient treatments.

**Methods:**

Study 1 used service utilization data from 191 individuals referred to the brief intervention at a single community health site in a metropolitan health service. Proportions of individuals retained across the intervention and the referral pathways accessed following the intervention were examined.

Study 2 examined 67 individuals referred to the brief intervention across 4 different sites in metropolitan health services. A range of measures of symptoms and quality of life were administered at the first and last session of the intervention. Effect sizes were calculated to examine mean changes across the course of the intervention.

**Results:**

Study 1 found that 84.29% of individuals referred to the intervention attended at least 1 session, 60.21% attended 2 sessions or more and 41.89% attended 3 or more sessions. 13.61% of the sample required their care to be “stepped up” within the service, whereas 29.31% were referred to other treatment providers following referral to the intervention. Study 2 found a significant reduction in borderline personality disorder symptom severity and distress following the intervention, and an increase in quality of life. The largest reduction was found for suicidal ideation (*d* = 1.01).

**Conclusions:**

Brief psychological intervention was a useful step between acute services and longer-term treatments in this stepped model of care for personality disorder. Suicide risk and symptom severity reduced and quality of life improved, with only a small proportion of individuals requiring ongoing support from the health service following the intervention.

## Background

Personality Disorder is a high prevalence and often chronic mental health condition [1]. Although common aspects of the disorder such as suicidality, self-harm and affect dysregulation mean that individuals with personality disorder symptoms, particularly those with borderline personality disorder, may frequently present to acute services such as emergency departments and inpatient services [2–4]; research into treatment for the disorder has primarily focused on long term psychotherapeutic interventions [5, 6]. Previous studies have suggested that a step-down model of care may be helpful in providing care to individuals with personality disorders [7, 8]. This paper provides a preliminary investigation of a brief psychological intervention for people with personality disorders who present in crisis to health services. The brief intervention is examined as one step in a stepped whole of service model of care for personality disorders, and this paper provides an evaluation of service usage and symptom change over two studies.

Personality disorder at its core includes difficulties relating to others and difficulties with a sense of self, both of which impair psychosocial functioning, and may present in a variety of ways [9, 10]. Personality disorders are a relatively high prevalence presentation within public mental health systems [4]. Although the estimated prevalence within the general population is 2.7–14.8% [11–13]; prevalence estimates of personality disorders in outpatient and inpatient samples are much higher, ranging up to 92% in outpatient services [14] and up to 66% in inpatient services [15]. Many health services have traditionally offered two levels of intervention for people with personality disorders: acute services (such as inpatient units, emergency departments and acute care teams located in outpatient settings) to manage escalations in risk, and long term intensive psychotherapy options [8]. However, individuals with personality disorders often face a range of barriers to accessing these longer-term treatment options including financial barriers, lack of understanding on how to access treatment or what services are available, long waitlists for treatment, and a lack of treatment options available depending on geographic area and service resources [16].

Although much research has focused on conditions such as anxiety and depression [17–19], there is growing interest in examining whether stepped care may be efficacious in the treatment of complex mental health conditions previously thought to require intensive treatment such as personality disorder [20, 21]. Stepped models of care [22] aim to match the level of intervention with the needs of an individual’s presenting issues, and are built on the principle of beginning with low intensity interventions before referring the individual to interventions of increasing intensity (also referred to as “stepping up”) if required [23, 24]. This model of care has been described as “self-correcting” as monitoring of an individual’s presenting symptoms and response to treatment is used to inform how an individual progresses through the “steps” of evidence based treatment available [22].

**Fig. 1:**
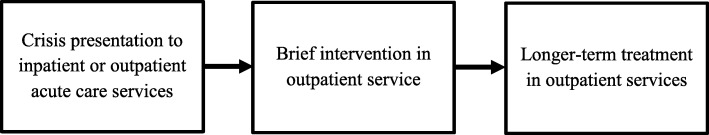
Illustration of brief intervention as part of a stepped model of care

There are several factors that need to be considered in applying a stepped care model effectively. These include the co-morbidity in mental health presentations, whether people need a diagnosis to access treatment for their symptoms, the fluctuations of symptoms and, by extension, level of intervention required over time, and whether a “watchful waiting” step is appropriate for all models [25]. These factors are especially important considerations for personality disorders which are associated with high rates of co-morbidity [26], challenges in diagnosis [27], and represent a significant proportion of mental health presentations to emergency departments and inpatient admissions [4]. A step-down approach to treatment has been proposed to address common challenges of providing adequate care to personality disorder clients [8]. This model includes short-term interventions of up to four sessions for individuals with personality disorders presenting in crisis (See Fig. 1). The aim is to reduce symptom severity in the short term and alter an individual’s trajectory through mental health care services, by reducing engagement with acute support services, and acting as a step towards longer-term psychotherapeutic or auxiliary supports. Contrary to other brief interventions, which often aim to condense a standard treatment course [28], a brief intervention for personality disorder in the context of step-down care does not seek to fully resolve an individual’s personality disorder symptoms. Rather, it aims to be a therapeutic step in their path to recovery by assisting an individual with managing their current crisis, before then assisting them to access other treatment options. Research is beginning to explore treatment dosage and the efficacy of briefer interventions for personality disorder, particularly in treating borderline personality disorder symptoms [20, 21, 29, 30], however, as outlined by Paris [20], many of these interventions are approximately six months in length. Relatively little research has explored treatment options shorter than this time frame or focused on crisis interventions. This is particularly notable, as many individuals with personality disorders may repeatedly access acute services such as emergency departments and inpatient services in the context of crisis, such as self-harm, suicidality or heightened emotion dysregulation [31, 32]. Evidence based interventions for crisis presentations to acute services have targeted the reduction of specific symptoms, such as suicide prevention [33], rather than targeting specific mental health conditions.

Brief crisis interventions for individuals with personality disorders or associated symptoms (predominantly self-harm and suicidal ideation) have primarily focused on pharmacotherapy to reduce arousal, or improving psychotherapeutic engagement [32], with mixed results. For example, in their recent trial of a volitional help sheet intervention for individuals presenting with deliberate self-harm, O’Connor et al. [34] found no significant differences in re-presentation rates for individuals in the intervention and treatment as usual conditions. Other brief interventions such as that trialled by Berrino et al. [35] have been located in an inpatient setting, and aimed at acute suicidal presentations. Although suitable for acutely unwell individuals, current guidelines recommend that treatment should occur within community-based outpatient settings [36–38].

There have been promising findings in examinations of brief community-based treatments. The “Green Card” model trialled by Wilhelm et al. [39] found that offering 3 sessions of structured therapy following a deliberate self-harm presentation led to a significant reduction in depression symptoms, and the majority of individuals made some lifestyle changes at follow-up 3–15 months post-intervention. In addition, individuals with co-morbid personality disorder and substance misuse who attended the six-session Manual Assisted Cognitive Therapy (MACT [40];) were found to have lower suicidal ideation, depression and anxiety than a treatment as usual condition at three-month follow-up.

A limitation of previous research examining brief intervention for personality disorder is that many studies do not examine how the intervention may operate in relation to other treatment options and services offered within an area. The provision of a brief intervention as part of a stepped model of care may allow services to make treatment more accessible for people with personality disorders and assist them to access treatment options which may in turn, reduce the frequency of their engagement with crisis services. Grenyer et al. [7], found that having a brief intervention for personality disorder available within a stepped care model allowed services to reduce the length of inpatient stays and number of emergency department presentations saving USD$2720 per patient per year.

The brief intervention used within the whole of service model [7] was a generalist, manualized psychotherapy to address the needs of individuals with personality disorders who presented to emergency care in crisis with high-risk and complex needs [41]. The intervention consists of up to four weekly sessions of therapy (detailed in Table 1), and was developed in line with recommended best practice for personality disorder treatment and a relational model of care [42, 43]. The model of treatment was informed by the “Green Card Clinic” [39], and was adapted for personality disorder presentations and to be applicable to different mental health care settings including youth and adult patients. As outlined in Table 1, the intervention is structured and combines care planning, skills-based intervention, and relational principles. In addition, it included engagement with the individual’s family, partner or carer in treatment and recovery planning as a specific planned part of the intervention.

**Table 1:** Session Objectives and Suggested Outline of the Brief Intervention Sessions

Session	Session Objectives	Session Outline
1	• Focus on developing rapport and a positive therapeutic relationship • Explore factors that led to the crisis • Begin to develop a Care Plan • Conduct a risk assessment • Provide psycho-education • Connect with carers	1. Build rapport and focus on developing a positive therapeutic relationship (throughout the sessions) 2. Set the frame for treatment (i.e. discuss the duration of the current and future sessions including the four session intention) 3. Provide information on the purpose of the clinic 4. Understand what led to the client’s crisis and provide a space for them to talk 5. Begin to develop a Care Plan, focusing on the ‘My crisis survival strategies’ section 6. Conduct a risk assessment 7. Provide client with psycho-education 8. Connect with the carers 9. Discuss need, and ascertain willingness, for further appointments 10. Encourage the client to think more about their values and goals
2	• Further engage the client • Understand the client’s goals and values • Further develop the Care Plan • Provide further psycho-education and support	1. Engage the client further 2. Discuss further the client’s goals and values 3. Develop the Care Plan further, focusing on ‘My main therapeutic goals and problems I am working on’ section 4. Provide an opportunity for the client to discuss any other issues 5. Provide psycho-education about the development and maintenance of specific problems 6. Conduct a risk assessment 7. Encourage the client to think about their plans after the clinic sessions are complete in between appointments and flag this to discuss further in Session Four 8. Provide psycho-education on the benefits of longer-term treatment for people with more enduring problems
3	• Focus on connection, assessment of needs and education • Allow the carer space to voice their concerns and needs • Assess the current needs of the carer and draft a Carer Plan with the carer for their needs • Provide information and education regarding mental illness, personality disorders, self-care and navigating the mental health system • Provide further referrals to more intensive family and carer interventions or other services	1. Set the frame of the session including the aims, purpose and confidentiality issues 2. Build rapport and focus on the needs of the carer 3. Assess the carers current needs and responses to the client’s recent crises and provide a space for them to talk 4. Develop a Carer Plan with the carer for their own self-care (see: Carer Plan) 5. Provide information and education regarding mental illness, personality disorders, self-care and navigation of the mental health system including who to call upon in a crisis 6. Discuss need, and ascertain willingness, for referral to family and carer services.
4	• Discuss the client’s plans for the future • Provide information on treatment options • Finalise the Care Plan and discuss relapse prevention • Provide referral to other services	1. Discuss further the client’s future plans 2. Consider and discuss treatment options 3. Finalise the Care Plan, focusing on ‘My support people’ section, and relapse prevention strategies 4. Link the client with other services, and provide referral where necessary

*Note.* Objectives and outline adopted from the intervention manual (Project Air Strategy for Personality Disorders, 2015)

### The current study

Within the whole of service model [7] the brief intervention is a step in the journey of an individual through the mental health service. It is an accessible treatment option to people presenting to acute mental health services in crisis to facilitate referral to longer term treatment options, and aims to significantly reduce the severity of presenting symptoms (although these are not expected to fully resolve within the timeframe of the intervention). This paper aimed to expand on previous research examining whole of service use and cost benefit analyses following the implementation of the intervention, by examining service use and symptom change during the course of the intervention within a whole of service stepped care model [7].

Preliminary evaluation of the intervention using both service-level and individual-level data is presented over two studies which aim to examine retention rates and referral pathways from the intervention, as well as symptom changes during the course of the intervention, in line with StaRI and STROBE guidelines (see Additional files). Study 1 used service utilization data to explore participant retention during the intervention and referral pathways following the intervention; in particular, how many individuals required an escalation of care within the public mental health service during or following the intervention. Study 2 examined the effectiveness of the intervention in reducing individual mental health symptoms and improving quality of life.

## Study 1: referral pathways and intervention retention

### Methods

Anonymized health service administrative data was analysed to evaluate the implementation of the brief intervention for one service. De-identified service use data was provided to researchers, thus did not involve explicit written consent procedures. The sample was comprised of all individuals referred to the brief intervention service over the course of the study. Data available for analyses consisted of referring mental health team, referral date, attendance at brief intervention sessions, and discharge plan. This information allowed evaluation of referral pathways, the time between referral and first appointment, and the total number of sessions attended. Use of this data was approved by the institution’s Ethics Committee and the health service’s Research Governance Committee and Records Manager.

#### Setting

This brief intervention was established and delivered in a metropolitan Australian community mental health outpatient service which utilizes a whole of service model for personality disorder treatment [7]. The community it serves is approximately 218,000 people [44] and has a single hospital mental health unit servicing individuals of varying acuity, along with associated community outpatient services available. The median age for this area is 40 years, 77.7% are Australian born, and 83.1% speak only English at home, with slightly more females (51.2%) than males (48.8%).

#### Intervention

Mental health staff, including psychologists, social workers, nurses and occupational therapists, administered the manualized brief intervention outlined in Table 1. Staff were trained in the administration of the manualized intervention prior to administering the intervention. Sessions were provided weekly for 50 min. Session plans were collaboratively developed between the clinician and individual and only deviated from the outline in Table 1 if clinically indicated. The third session for families and carers required the cooperation and consent of the individual to identify those relevant to attend, and could be conducted by phone with the provision of educational materials if face-to-face attendance was not feasible. Referrals following the intervention, and escalations in care during the intervention (e.g., admission to inpatient units) were determined using clinical judgement of the clinician in consultation with the treatment team where required.

Individuals were eligible for the intervention if they were aged 18 years or older and presented with suicidal thoughts or plans, recent episodes of self-harm behaviours or suicide attempts, emotion dysregulation, and/or a personality disorder. Individuals were not considered for the intervention if there was evidence of psychosis, alcohol or other drug dependency as a primary presenting issue, or if the level of risk identified was marked urgent under the relevant Mental Health Triage Policy.

#### Statistical analyses and coding

Proportions of individuals accessing the intervention, attending each session, and accessing different referral pathways following the intervention were calculated. Referral pathways into and out of the intervention were independently coded by two authors (inter-rater reliability = .98). This was done to examine the use of the intervention including attrition during the intervention, the proportion of individuals requiring stepped up care within the public health service, and the proportion of individuals referred out of the service to other treatment providers.

Referral pathways out of the intervention were coded into six categories. First, “intervention unsuitable” denotes cases where the treating clinician assessed the intervention to be unsuitable for the individuals presenting needs or living situation. This occurred where the individual either 1) did not meet the eligibility criteria for the intervention or 2) lived outside of the health district and was referred on to their local service. Second, “referred up” denotes individuals whose needs were assessed by clinicians as requiring their care to be stepped up within the health service either to manage risk (e.g., requiring an inpatient admission) or to receive a longer psychological intervention such as group or individual therapy. Third, “referred out” denotes individuals who were referred on to treatment options outside of the public health service such as physicians (general practitioners [GP]), private psychology, or Non Government Organisations (NGO). Individuals who discontinued the intervention were identified as either “lost to service,” indicating that they had not attended a session and were not able to be contacted, or “withdrew” indicating that the individual did not attend further sessions and information available indicates that the individual discussed this choice with their clinician. Finally, individuals where no information was available regarding their referral pathway were coded as “unknown”.

## Results

### Origin of referral and attendance

During the 7 year study period, 191 individuals were referred to the brief intervention for treatment. Origin of referral was recorded for 147 individuals. The majority of referrals to the brief intervention (*n* = 140, 95.23%) were referred through the mental health service Acute Care Team (an outpatient mental health service for individuals in crisis), and the remaining were referred from the hospital’s mental health inpatient unit (*n* = 7, 4.76%). Individuals were offered their first appointment upon referral, and of 171 individuals with recorded dates, 80.12% (*n* = 137) were offered their appointment within 7 days of referral (median = 3, range = 0–45). Of those referred to the brief intervention clinic, 84.29% (*n* = 161) attended at least one session, 60.21% (*n* = 115) attended two or more sessions, and 41.89% (*n* = 80) completed all three individual sessions. The optional carer/family session was consented to by 6.28% (*n* = 12) of individuals. Thirty of the 191 referred clients (15.71%) did not attend any brief intervention sessions following referral.

### Referral pathways following the intervention

**Fig. 2:**
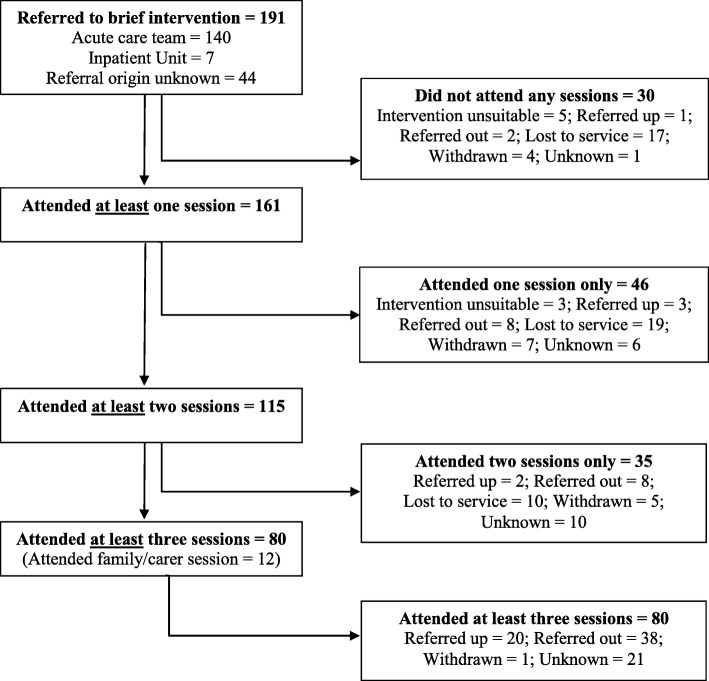
Flow chart of participants referred to the brief intervention, sessions attended and outcome

Movement through the brief intervention is illustrated in Fig. 2. Discharge plan information was available for 103 individuals who had participated in the brief intervention, and non-attendance information was available for 115 individuals. Outcome was unknown for 38 individuals. Overall, only a small percentage (2.62%, *n* = 5) of referrals were not appropriate for the brief intervention. An additional three people (1.57%) moved out of the health district following their initial session and did not continue (coded as “intervention unsuitable” in Fig. 2). Individuals who became lost to service made up a large proportion (*n* = 17, 56.67%) of people who did not attend any sessions; however, they made up a smaller proportion (*n* = 29, 18.01%) of people who discontinued the intervention. Overall, 8.90% (*n* = 17) withdrew either prior to or during the intervention. People who withdrew identified several reasons for discontinuing the intervention including re-engaging with other supports (such as private psychology, *n* = 4), work commitments (*n* = 3), resolution of the crisis (*n* = 3) and declining further sessions (*n* = 6).

Individuals who required a higher level of care within the public health system and were “referred up” made up 13.61% (*n* = 26) of the sample. Of note, only three (1.57%) individuals were referred up to an inpatient admission during the course of the intervention. The majority of individuals referred up within the service (*n* = 23, 12.04% of individuals referred to the brief intervention) were referred to ongoing treatment within outpatient mental health services. The majority (*n* = 21, 91.30%) of individuals referred for additional treatment within community mental health completed three or more sessions of the intervention. All 23 were referred for either individual or group psychological interventions within the community mental health service, and five were also referred to additional support outside of the health service (e.g. private psychiatry, GP, domestic violence counselling).

Overall, 29.31% (*n* = 56) of individuals were referred out of the service to other treatment providers. Of this group, 26.79% (*n* = 15) were referred on for psychological treatment only, 42.86% (*n* = 24) were referred to a GP only (this referral may have facilitated a mental health treatment plan and psychology referral, or medication), and 30.36% (*n* = 17) were referred to a combination of medical and psychological treatment (i.e., generalised medical and psychological treatment, or specialised medical and psychological treatment). Referrals were made to a broad range of treatment services including NGOs, private psychology, university counselling services, alcohol and other drugs programs, and parenting programs.

## Discussion

Study 1 found that the brief intervention was a useful conduit between acute levels of care (inpatient, acute care teams) and longer-term options, consistent with a step-down model of care [8]. Of note, only 1.57% of individuals required an inpatient admission during the intervention, and 12.04% had their care stepped up within the public health service following the intervention. The variety of services and treatment referrals following the intervention may reflect the heterogeneous nature of crisis presentations in individuals with personality disorders, and is consistent with a person-centred model of care. The retention rates across the course of the intervention were comparable to other short-term crisis interventions [39]. Although this study provides initial indications of how the intervention may function within an outpatient mental health service, it does not provide information about initial presentations or symptom change during the intervention. This was explored in Study 2.

## Study 2: symptom change during the brief intervention

### Methods

#### Participants

Participants were 67 public health service clients referred to one of four brief intervention clinics from their local hospital’s inpatient unit or emergency department in New South Wales, Australia. Participants were recruited as part of an ongoing longitudinal study [45], and were invited to participate by their health service clinician. Participation was voluntary and did not influence the treatment received. All participants with pre and post measures for the brief intervention were included in the study. Using available information (gender *n* = 65, age *n* = 59), 75.39% (*n* = 49) of the sample were female, and the mean age for the sample was 31.54 years (*SD* = 13.40, range = 18–68). Relationship status was recorded for 41 participants: 51.22% (*n* = 21) were single, 29.27% (*n* = 12) were in a relationship (including married and de facto), and 19.51% (*n* = 8) were divorced, separated or widowed. The study was approved by the institutions Ethics Committee and the relevant health district’s Research Governance Office. All participants provided written, informed consent to participate.

#### Measures

Participants gave approval for access to their electronic medical record, whereby demographic information was collected. Clinical information was collected using a self-report questionnaire designed to track symptom change during the course of the intervention, and was administered upon presentation to their first appointment, and during their last appointment. The questionnaire included measures of personality disorder symptoms, distress, quality of life, suicidal ideation and deliberate self-harm. A brief description of the measures follows.

##### Distress

The Mental Health Inventory 5 (MHI-5) [46] is a 5-item measure of distress, regularly used to screen for mental health, including depression and anxiety. The MHI-5 uses a 6-point Likert scale from 1 = *‘none of the time’* to 6 = *‘all of the time’.* The MHI-5 demonstrated adequate reliability in the current sample at both pre- (α = .84) and post- intervention (α = .87).

##### Personality disorder symptom severity

The severity of borderline personality disorder (BPD) symptoms as outlined in the *Diagnostic and Statistical Manual of Mental Disorders* (fifth edition, DSM-5) were rated 1 = ‘*none of the time’*, to 6 = ‘*all of the time’* to provide a dimensional understanding of symptom experience [45, 47]. The item wording, adapted from the McLean Screening Instrument for BPD (MSI-BPD, [48]), addresses the nine DSM-5 [9] criteria across 10 items. Each item addresses one criterion and two items assess the ninth criterion of stress-related paranoia and dissociation. The deliberate self-harm/suicide item of the MSI-BPD was asked as presence or absence, and participants were then asked to identify how many times in the past two weeks they had engaged in deliberate self-harm. Internal consistency of the nine dimensional items was acceptable at pre- (α = .80) and post-intervention (α = .78).

##### Suicidal ideation

Suicidal ideation was measured using the single item from the Beck Depression Inventory, as has been used successfully by others to examine suicidal ideation, with demonstrated adequate concurrent validity with other validated self-report and clinician administered measures of suicidal ideation [49, 50].

##### Quality of life

Quality of life was assessed using a global item (‘How would you rate your quality of life?’), measured on an 11-point Likert scale from 0 = ‘*very bad*’ to 100 = ‘*very good*’. Global measures such as this have been used extensively in health research [51], demonstrating good convergent validity [52].

#### Statistical analysis

To examine the presence of symptoms associated with BPD, a score of ≥2 on each item was considered as ‘present’. Changes over time on the MHI-5, DSM-5 BPD symptoms, quality of life and self-harm/suicidal ideation were analysed using within subjects repeated measures analysis for continuous variables and related samples McNemar’s change test was used for categorical variables. Cohen’s *d* effect sizes were calculated to determine degree of change on continuous variables [53].

## Results

The average number of ‘present’ DSM-5 symptoms at baseline was 7.55 (*SD* = 1.62, *n* = 60), suggesting this is a highly symptomatic sample. At baseline, 68.25% of participants had engaged in self-harm, a suicide attempt, or both in the two weeks prior. The mean quality of life rating was 37.34 (*SD* = 18.45). Following the intervention, clients experienced a significant improvement on all outcome measures (see Table 2). Effect sizes ranged from moderate to large [53], and were largest for suicidal ideation (*d* = 1.01), quality of life (*d* = .95) and scores on the MHI-5 (*d* = .79). There was also a significant reduction (McNemar *p* < .001) in the number of people who had self-harmed or attempted suicide in the two weeks prior (68.18% upon commencement of intervention, and 21.54% upon completion).

**Table 2:** Pre and post intervention scores on the DSM-5 symptoms, MHI-5, and quality of life measures

		Pre intervention		Post intervention		*t*	*p*	*d*
	n	M	SD	M	SD			
Total DSM-5 symptoms /9	60	7.55	1.62	6.43	2. 21	4.97	.000	.58
MHI-5 total	65	21.28	4.69	17.40	5.18	5.80	.000	.79
Quality of life	64	37.34	18.45	55.63	19.99	−6.85	.000	.95
BPD symptoms								
Unstable relationships	61	3.79	1.59	2.62	1.62	5.00	.000	.73
Impulsivity	63	3.79	1.37	2.87	1.37	4.96	.000	.67
Mood dysregulation	64	4.11	1.39	3.14	1.46	5.87	.000	.68
Anger	64	3.66	1.22	2.84	1.28	5.22	.000	.66
Paranoid ideation	64	3.75	1.23	3.17	1.20	3.48	.001	.48
Chronic Emptiness	65	4.05	1.32	3.22	1.46	4.54	.000	.60
Identity disturbance	65	3.25	1.60	2.75	1.49	2.87	.006	.32
Real or imagined abandonment	65	3.52	1.85	2.55	1.43	4.47	.000	.59
Self-harm and suicide attempt frequency (2 weeks)	63	2.16	3.35	.75	2.31	3.54	.001	.49
Suicidal ideation	63	2.30	.75	1.56	.70	6.94	.000	1.01

*Note. N* = 67, *n* indicates data available for that analysis

## Discussion

The findings of Study 2 indicate a significant reduction in general mental health symptoms, BPD symptoms and increase in reported quality of life following the intervention. The largest effect size was found for reduction in suicidal ideation, followed by increase in quality of life. The smallest change was found for identity; although significant at *p* = .006, this is unlikely to translate into practical changes as *d* < .41, the suggested minimum effect size for practical change [54]. This finding is in line with previous research, which indicates that difficulties with self or identity functioning may take time to improve in treatment [55–57]. Consistent with expectations, although there was a significant reduction in severity of BPD symptoms, the sample still reported clinically significant levels of symptoms with an average of 6.43 symptoms at the end of the intervention. Overall, these findings indicate that despite still experiencing BPD symptoms at the end of the brief intervention, participants experienced significant and meaningful reductions in their symptom severity and improvements in their quality of life.

## General discussion

This paper aimed to provide a preliminary investigation of a brief intervention for personality disorder, located as a step within a whole of service model of care that included acute and longer-term community treatment steps. Two preliminary studies were conducted to examine the utility of the intervention: one examining the referral paths and attendance at a single site, and one examining symptom change across the length of the intervention. Overall the findings of both studies suggest that the brief intervention for personality disorder was a useful step in care for individuals with personality disorders within this model.

The findings of Study 1 suggest high take up of the intervention amongst those referred with 84.29% attending one or more sessions. The attrition over time during the intervention was comparable with similar crisis interventions [39]. This drop-off during the intervention may be attributable to lessening distress within participants. Overall the pattern of attendance is consistent with previous research indicating high attendance of individual therapy sessions by people with personality disorders when distressed, and lower attendance when not distressed [58]. The proportion of individuals who did not attend the intervention, or only attended one session may have important implications for the provision of treatment services for personality disorders. Models of care where individuals are referred directly from crisis services to long term specialist treatments, may not consider this pattern of attendance. As such, providing an intermediate treatment step may help services to distribute treatment resources to more individuals by preserving more complex, longer term treatment services for those more able to engage at that point in their recovery.

In line with stepped care principles, only a small proportion of individuals who were referred to the clinic required additional treatment within the health service, and the majority of these individuals had this transition occur after first completing the brief intervention. In addition, only a small proportion (1.57%) of individuals required an inpatient admission following engagement with the brief intervention. The findings of Study 1 demonstrate that the brief intervention played an important role in transitioning between acute and longer-term treatment options for individuals with personality disorder. As such, the brief intervention acted as an important therapeutic step in a step-down model of care [8] following a crisis presentation.

The findings of Study 2 indicate a significant reduction in a range of symptoms during the course of the intervention. Overall, the strongest changes were for suicidal ideation, quality of life and distress. Although some part of this change may be attributable to an overall reduction in distress, which may partially inflate some responses [59], the effect sizes indicate that these changes are likely to reflect meaningful change in symptom presentations. The symptom with the least change during the course of the intervention was identity disturbance. This finding is consistent with previous theory and research, which indicates that these symptoms may be slow to change, and are likely to require further intervention [55–57]. Overall, these preliminary findings indicate that the brief intervention is effective at reducing distress and providing support following a crisis presentation.

The use of the brief intervention for personality disorder has previously been shown to lead to reductions in crisis service use, and cost-savings consistent with international estimates [7, 60]. The findings of Study 1 and 2 further support the use of brief intervention in treating personality disorder, as they indicate clinical effectiveness and that the intervention works as a mechanism of stepped care within a whole of service model. These findings have implications for the types of stepped care that could be provided to people with personality disorders. For example, the brief intervention could be a step prior to other short therapeutic interventions such as the 12-week skills intervention identified by Laporte et al. [21].

The findings of Study 1 and 2 may also have implications for the treatment of individuals with suicidality and self-harm, and the management of treatment resources in health services. Examination of treatment as usual approaches suggest that these approaches are associated with improvement in several clinical outcomes, however the effects are smaller for suicidality and self-harm [61]. In the context of these findings, the brief intervention may broaden options for care connection and planning for individuals with suicidality or self-harm, and may help to reduce waitlists for specialist programs.

There are a number of limitations to the studies presented. Although the findings of Study 1 and Study 2 are promising, they are limited by their small sample sizes, lack of baseline diagnoses, and use of self-report measures in Study 2. Further replication of these findings with a larger sample, and collection of diagnostic assessment data is required to understand the generalizability of these results. In addition, the assessment of adherence to the intervention manual was not performed, but is recommended for future outcome research. Information provided for this evaluation was naturalistic data from actual implementation in health care sites, and so did not have the systematic rigour in data collection and audit as would have occurred in a clinical trial research protocol.

Future studies should expand on Study 1 and examine whether there are gender, age, or cultural background related effects in referral and retention within the intervention. Study 1 also only examined inpatient admissions and referrals during the course of the intervention. The inclusion of longitudinal service use data post-intervention in future studies would allow for better understanding of re-presentation rates and inpatient admissions.

The take up of the family/carer session in Study 1 was quite low, and the data available did not capture other forms of support for family members used in the health service such as phone calls, provision of education materials for individuals to pass on to their family members, and referring families to longer term support options. In addition, staff attitudes and skills in working with families were not measured during the evaluation and it is unknown whether these factors could have contributed to uptake. Future research should further examine the take up of the family/carer session and support provided to carers and family members as an adjunct to treatment for people with personality disorders.

Interpretation of the findings of Study 2 is limited by the lack of a comparison group and future studies should include a treatment as usual condition to explore how much of the change seen in Study 2 is the result of the intervention, and how much may be due to other factors such as time since the crisis presentation. Finally, the findings from Study 2 are from a single site, and may not all be applicable to other treatment services. Examination of the intervention across a range of sites is required in future studies.

## Conclusions

A brief intervention for people with personality disorders was found to be an effective step within a whole of service model of care. The findings of Study 1 indicate retention of individuals within the intervention are comparative to related approaches, and that the intervention may act as a treatment step linking acute responses to longer term treatment options for individuals with personality disorder. The findings of Study 2 indicate a significant reduction in distress as well as self-harming and suicidal ideation following the intervention.

## Data Availability

The data that support the findings of Study 1 are available from NSW Ministry of Health but restrictions apply to the availability of these data, which were used under license for the current study, and so are not publicly available. Data are however available from the authors upon reasonable request and with permission of NSW Ministry of Health. Applications to access the data may be sent to NSW Ministry of Health, Locked Mail Bag 961 North Sydney 2059, Australia. Email: MOH-MentalHealthBranch@health.nsw.gov.au The data that support the findings of Study 2 are available on request from the corresponding author BG. The data are not publicly available as they contain information that could compromise research participant privacy and consent.

## Abbreviations

BPD: Borderline Personality Disorder
DSM-5: Diagnostic and Statistical Manual of Mental Disorders, Fifth Edition
GP: General Practitioners
MACT: Manual Assisted Cognitive Therapy
MHI-5: Mental Health Inventory 5
MSI-BPD: McLean Screening Instrument for BPD
NGO: Non-Government Organisations
StaRI: Standards for Reporting Implementation Studies
STROBE: Strengthening the Reporting of Observational Studies in Epidemiology
